# Efficacy and safety of efgartigimod in very-late-onset myasthenia gravis

**DOI:** 10.3389/fimmu.2026.1808357

**Published:** 2026-04-13

**Authors:** Yunbin Zhao, Meijie Qu, Xi Rong, Xupeng Sun, Li Wang, Min Liu, Jiaona Sui

**Affiliations:** Department of Neurology, The Affiliated Hospital of Qingdao University, Qingdao, China

**Keywords:** acetylcholine receptor, conventional immunosuppressants, efgartigimod, myasthenia gravis, neonatal Fc receptor antagonist, very-late-onset myasthenia gravis

## Abstract

**Background:**

Myasthenia gravis (MG) is an autoimmune disease. Recently, the prevalence of very late-onset MG (VLOMG; onset ≥ 65 years) has rapidly increased. The treatment of VLOMG groups is associated with several challenges, including multiple comorbidities, polypharmacy, and immunosenescence. Efgartigimod, which is the first approved neonatal Fc receptor (FcRN) antagonist, is reported to rapidly ameliorate MG symptoms with a favorable safety profile. Thus, efgartigimod is a potential novel therapeutic for VLOMG.

**Objective:**

This study retrospectively analyzed the clinical data of patients with VLOMG treated with efgartigimod at our center over the past two years to assess its long-term efficacy and safety.

**Methods:**

This study retrospectively enrolled 62 patients with VLOMG who received at least one efgartigimod treatment cycle. The primary efficacy outcomes were the proportion of patients achieving minimum symptom expression (MSE) and the reduction in glucocorticoid dosage. The secondary efficacy outcomes were changes in Myasthenia Gravis-Activities of Daily Living (MG-ADL) scale scores, Quantitative Myasthenia Gravis (QMG) scale scores, Myasthenia Gravis Quality of Life 15-item revised (MG-QoL15r) scale scores, and scores of key muscle groups (ocular, bulbar, respiratory, axial, and limb muscles) relative to baseline.

**Results:**

This study enrolled 62 eligible patients with VLOMG. Of these 62 patients, 37, 10, and 15 completed 1, 2, and ≥3 treatment cycles, respectively. The cumulative MSE achievement rate was 41.9% (26/62) by the end of the study. MG-ADL, QMG and MG-QoL15r scores after treatment were significantly reduced relatively to baseline in patients who underwent 1, 2, and ≥ 3 treatment cycles (all P < 0.001, P < 0.01, and P < 0.05, respectively). Subgroup analysis of muscle groups revealed that bulbar, limb (including axial), and ocular muscle function after treatment was significantly improved relatively to baseline in 37 patients (all P < 0.01). Limb (including axial) muscle function after treatment was significantly improved relatively to baseline in 15 patients (P < 0.05). Glucocorticoid dosage after treatment was significantly decreased relatively to baseline in patients who underwent 1, 2, and ≥ 3 treatment cycles (P < 0.0001, P < 0.05, P < 0.05, respectively). During treatment with efgartigimod, one patient developed an allergic rash, one patient experienced gastric discomfort, and two patients reported headache.

**Conclusion:**

This study demonstrated that efgartigimod effectively ameliorates symptoms and improves the quality of life in patients with VLOMG and enables short-term glucocorticoid dosage reduction. Thus, efgartigimod seems to be a safe and effective long-term therapeutic agent for VLOMG.

## Introduction

Myasthenia gravis (MG) is a T cell-dependent autoimmune disease in which B cells produce autoantibodies targeting the acetylcholine receptor (AChR) on the postsynaptic membrane of the neuromuscular junction. Several pathogenic autoantibodies are detected in patients with MG. The most common pathogenic antibody in MG is the AChR antibody, followed by muscle-specific kinase (MuSK) and lipoprotein receptor-related protein 4 (LRP-4) antibodies. The pathogenic mechanisms of AChR antibodies involve the binding to AChR to block its function, inducing AChR internalization and degradation, and mediating postsynaptic membrane damage via the complement system (1–4). Therapeutics targeting different pathogenic pathways are available for patients with AChR antibody-positive MG, including C5 complement inhibitors (complement pathway), FcRN antagonists (antibody reduction pathway), and B-cell antagonists (antibody production inhibition pathway) (5).

Based on the clinical and therapeutic characteristics, patients with MG are categorized into the following three subgroups: early-onset MG (EOMG; onset < 50 years), late-onset MG (LOMG; onset ≥ 50 years to < 65 years), and very late-onset MG (VLOMG; onset ≥ 65 years) groups. The prevalence of LOMG and VLOMG has rapidly increased. Patients with LOMG and VLOMG are characterized by a high burden of comorbidities (such as infection, hypertension, diabetes mellitus, osteoporosis, and malignant tumors), polypharmacy, and immunosenescence (such as impaired immune function, inflammaging, and increased autoantibody production) (6, 7). The management of comorbidities and drug adverse effects is a major clinical challenge (8). Compared with patients with LOMG, patients with VLOMG exhibit poor tolerance to the side effects of MG therapeutics and are consequently associated with higher treatment risks. While conventional treatment modalities for MG, including thymectomy, corticosteroids, intravenous immunoglobulin, plasma exchange, and non-specific immunosuppressants, have significantly reduced mortality, they often fail to achieve complete disease control, leaving many patients with disease symptoms or adverse drug reactions (9). Additionally, comorbidities in elderly patients (such as cerebrovascular disease, diabetes mellitus, chronic renal failure, and osteoporosis) influence treatment selection and increase the susceptibility to drug side effects (10, 11). Thus, there is an urgent need to develop novel therapeutic strategies for patients with VLOMG.

Recently, several novel biological targeted agents have been approved for MG treatment. These agents exhibit excellent efficacy and a favorable side effect profile. Efgartigimod is an Fc fragment of a human IgG1 antibody engineered to bind to the neonatal Fc receptor (FcRN). Multiple mutations (M252Y/S254T/T256E/H433K/N434F) are introduced at the CH2-CH3 domain junction of the Fc region through ABDEG technology. These mutations enhance the affinity of the antibody for FcRN at both neutral and acidic pH, inhibit the interaction between FcRN and IgG, reduce IgG recirculation, and promote IgG degradation (12, 13). Similarly, Rozanolixizumab, a newly approved humanized IgG4 monoclonal antibody targeting human FcRn for the treatment of MG, offers broader applicability than efgartigimod—it was the first drug approved in the United States for generalized MG in patients positive for both AChR and MuSK antibodies. It has also demonstrated favorable outcomes in clinical trials for MG, further supporting the therapeutic potential of FcRn blockade (14). Plasma exchange-related studies have demonstrated that the downregulation of circulating IgG levels effectively ameliorates symptoms (15). In the phase III ADAPT clinical trial, efgartigimod exhibited a rapid onset of action and alleviated symptoms in patients with MG. The mean age of patients in the efgartigimod treatment group was 45.9 ± 14.4 years (maximum age: 78 years). In multicenter real-world cohort studies in Japan and China, most patients treated with efgartigimod rapidly achieved clinically meaningful improvements (CMIs). This suggests that efgartigimod is a potential therapeutic for LOMG (16, 17). Current studies on efgartigimod in VLOMG are mainly real-world observations and have preliminarily confirmed its efficacy and safety. However, these studies are associated with several limitations, such as small sample sizes (12 and 15 cases in two studies) (18, 19) and the lack of long-term follow-up. Therefore, further studies with large sample sizes are needed to evaluate the long-term efficacy and safety of efgartigimod in VLOMG. It is worth mentioning that efgartigimod has been included in China’s national medical insurance, but it is not fully reimbursed. For example, the employee medical insurance reimburses approximately 80%, while the resident medical insurance reimburses about 50%. As a result, the out-of-pocket expenses remain a significant financial burden for some patients.

This study aimed to examine the efficacy and safety of efgartigimod in VLOMG, especially its long-term therapeutic effects, by retrospectively analyzing the clinical data of patients with VLOMG treated at our center over the past two years.

## Methods

### Methods

#### Study design, participants, and data collection

This retrospective study analyzed the clinical data of patients with MG who underwent at least one efgartigimod treatment cycle at The Affiliated Hospital of Qingdao University from November 2023 to September 2025. The patients were grouped based on the number of treatment cycles they completed: 1 cycle, 2 cycles, or ≥3 cycles. The study was approved by the Ethics Committee of The Affiliated Hospital of Qingdao University (Approval No.: QYFYWZLL30692). All clinical data was collected from medical records and face-to-face follow-up interviews after obtaining written informed consent from all patients.

Inclusion criteria: The inclusion criteria were as follows: patients with a confirmed diagnosis of MG with positive AChR antibodies; patients who underwent at least one efgartigimod treatment cycle; patients aged ≥ 65 years at disease onset.
Exclusion criteria: The exclusion criteria were as follows: patients who received other biological agents, intravenous immunoglobulin (IVIg) or therapeutic plasma exchange (TPE) 6 months before enrollment; patients with incomplete baseline and follow-up data; patients with pre-existing minimum symptom expression (MSE) status at enrollment.

The following baseline clinical data were collected: age, gender, disease duration, comorbidities (such as hypertension, diabetes mellitus, and thymoma), follow-up duration, baseline quantitative MG (QMGs) score, MG-activities of daily living (MG-ADL) scale score, MG quality of life 15-item revised (MG-QoL15r) scale score, Myasthenia Gravis Foundation of America (MGFA) clinical classification, key muscle group function, anti-AChR antibody titer, proportion of patients achieving MSE, prior medication use before efgartigimod treatment, number of efgartigimod treatment cycles, and reasons for not completing ≥ 3 efgartigimod treatment cycles. The primary endpoints were the MSE achievement rate after 1, 2, and ≥ 3 efgartigimod treatment cycles. The secondary endpoints were changes in MG-ADL, QMG, MG-QoL15r scores, and key muscle group function and dosage reduction of conventional immunotherapies (such as glucocorticoids). Patients who did not complete ≥ 3 treatment cycles were followed up continuously after treatment discontinuation with documentation of medication dosage, MG-QoL15r scores, and adverse events.

#### Treatment and follow-up protocol, outcome measurement

All patients received intravenous infusions of efgartigimod (10 mg/kg body weight) once weekly for 4 consecutive weeks, followed by a 4-week interval with repeated cyclic administration thereafter. Concomitant basic treatments included pyridostigmine bromide, glucocorticoids, and immunosuppressants (mycophenolate mofetil and tacrolimus) with dosage adjustments or discontinuation based on disease progression. Follow-up was conducted once weekly during the first treatment cycle and once monthly thereafter. Standardized face-to-face clinical assessments were performed using a unified questionnaire at each follow-up to collect the following key efficacy indicators: MSE: (MG-ADL score of 0 or 1) (20); MG-ADL scale score: an 8-item patient-reported scale with a total score ranging from 0 to 24 (higher scores indicate more severe MG); a reduction of ≥ 2 points from baseline was defined as CMI; QMG score: a 13-item scale evaluating 5 major muscle groups with a total score ranging from 0 to 39 (higher scores indicate more severe MG); a reduction of ≥ 3 points relative to baseline was defined as CMI; MG-QoL15r scale score: a self-reported questionnaire assessing mental health and social function in patients with MG, consisting of 15 items across 4 dimensions with a total score ranging from 0 to 60 (a score of 0 indicates the best quality of life, while that of 60 indicates the highest impairment).

### Statistical analysis

Descriptive statistics were used to analyze baseline variables. Categorical variables are presented as frequencies and percentages. Normally distributed continuous variables are presented as mean ± standard deviation. Non-normally distributed continuous variables are presented as median and interquartile range (IQR). Repeated measures data were compared using repeated measures analysis of variance, followed by a Bonferroni *post-hoc* test. Paired t-tests were used for comparing normally distributed data (verified by the Shapiro-Wilk test), while nonparametric tests (Wilcoxon signed-rank test) were used for comparing non-normally distributed data. Chi-square test and McNemar test were used for proportional data. Differences were considered significant at P < 0.05. All statistical analyses were performed using IBM SPSS Statistics 26.0.

## Results

### Patient characteristics

From November 2023 to September 2025, 1108 patients with MG, including 357 patients with VLOMG, were treated at The Affiliated Hospital of Qingdao University. Of these, 84 met the enrollment criteria. Among these 84 patients, 22 were lost to follow-up. Thus, the final analysis was performed with the data of 62 patients with VLOMG. Of these 62 patients with VLOMG, 37, 10, and 15 received 1, 2, and ≥ 3 efgartigimod treatment cycles, respectively. Among all 62 patients, the median follow-up duration and baseline age were 14.00 (7.00–16.00) months and 72.00 (68.75–76.00) years, respectively. The clinical characteristics of the study cohort were as follows: male proportion: 53.2%; anti-AChR antibody-positive: 100%; comorbidities(79.0), hypertension, (48.4%), diabetes mellitus (30.7%), and cerebrocardiovascular disease (21.0%); baseline MG-ADL score: 5.29 ± 2.88; baseline QMG score: 13.19 ± 5.12; MG-QoL15r score: 19.71 ± 10.83; MGFA clinical classification: Grade II (51.6%), Grade III (27.4%), and Grade IV (21.0%) (**Table 1**); concomitant MG treatments: cholinesterase inhibitors (82.3%), tacrolimus (74.2%), glucocorticoids (51.6%) and mycophenolate mofetil (43.5%). The median number of efgartigimod treatment cycles was 1.00 (1.00–2.00). The main reasons for not completing ≥ 3 cycles were voluntary discontinuation due to economic factors after achieving MSE (n = 19) or significant symptom improvement (n = 15), with switching to conventional immunosuppressant therapy accounting for 34/47 (72.3%) of such cases. Discontinuation due to adverse events (rash) accounted for 2.1% of the cases (1/47) (**Table 2**).

**Table 1:** Patient characteristics at efgartigimod initiation.

Characteristic	1 cycle (n = 37)	2 cycles (n = 10)	≥ 3 cycles (n = 15)	All MG patients (n = 62)
Age, years, median (IQR)	72.00 (68.00–75.00)	73.50 (71.25–76.00)	70.00 (67.50–74.00)	72.00 (68.75–76.00)
Sex, n (%)				
Male	19 (51.4)	4 (40.0)	10 (66.7)	33 (53.2)
Female	18 (48.7)	6 (60.0)	5 (33.3)	29 (46.8)
Duration of MG, Months, median (IQR)	12.00 (2.00–30.00)	12.00 (0.95–46.50)	40.00 (4.00–60.00)	12.00 (2.00–41.50)
Complications, n (%)				
Thymoma	3 (8.1)	1 (10.0)	1 (6.7)	5 (8.1)
Thymectomy	2 (5.4)	–	–	2 (3.2)
Hypertension	15 (40.5)	6 (60.0)	9 (60.0)	30 (48.4)
Diabetes	10 (27.0)	3 (30.0)	6 (40.0)	19 (30.7)
Cardiovascular and cerebrovascular diseases	8 (21.6)	2 (20.0)	3 (20.0)	13 (21.0)
Malignant tumor (except for thymoma)	–	1 (10.0)	1 (6.7)	2 (3.2)
Other complications	10 (27.0)	5 (50.0)	8 (53.3)	23 (37.1)
Score (mean ± SD)				
ADL	5.03±2.43	4.70±2.67	6.33±3.85	5.29±2.88
QMG	13.95±5.27	11.20±4.83	12.67±4.82	13.19±5.12
QoL 15r	18.92±10.16	19.20±9.54	22.00±13.40	19.71±10.83
MGFA, n (%)				
II	15 (40.5)	6 (60.0)	11 (73.3)	32 (51.6)
III	12 (32.4)	2 (20.0)	3 (20.0)	17 (27.4)
IV	10 (27.0)	2 (20.0)	1 (6.7)	13 (21.0)
Key muscle groups, median (IQR)				
Respiratory muscles	0.00 (0.00–0.00)	0.00 (0.00–0.00)	0.00 (0.00–0.00)	0.00 (0.00–0.00)
Bulbar muscles	1.00 (0.00–2.00)	0.50 (0.00–1.00)	0.00 (0.00–2.50)	1.00 (0.00–2.00)
Limb muscles and axial muscles	8.00 (6.00–12.00)	5.00 (4.25–10.00)	7.00 (6.50–10.00)	8.00 (5.25–10.75)
Extraocular Muscles	3.00 (3.00–4.00)	3.00 (3.00–5.00)	3.00 (1.50–6.00)	3.00 (3.00–5.00)
Anti-AChR antibody level, nmol/L, n (%)				
< 10	8 (21.6)	2 (20.0)	3 (20.0)	13 (21.0)
10–20	13 (35.1)	4 (40.0)	6 (40.0)	23 (37.1)
> 20	16 (43.2)	4 (40.0)	6 (40.0)	26 (41.9)

IQR, interquartile range; SD, standard deviation.

**Table 2:** Patient condition during treatment with efgartigimod.

Treatment characteristics	All MG patients (n = 62)
Conventional immunotherapy, n (%)	
Pyridostigmine bromide	51 (82.3)
Glucocorticoids	32 (51.6)
Tarolimus*	46 (74.2)
Mycophenolate mofetil	27 (43.5)
Number of treatment cycles, cycle, median (IQR)	1.00 (1.00–2.00)
Reason for treatment being less than 3 cycles, n (%)	47 (75.8)
Achieve MSE or improvement	34 (72.3)*
Symptoms worsen	5 (10.6)*
No significant improvement	7 (14.9)*
Adverse reactions	1 (2.1)*

IQR, Interquartile range; *indicates the proportion among the 47 patients who received less than 3 cycles of treatment.

### MSE achievement rate

**Figure 1:**
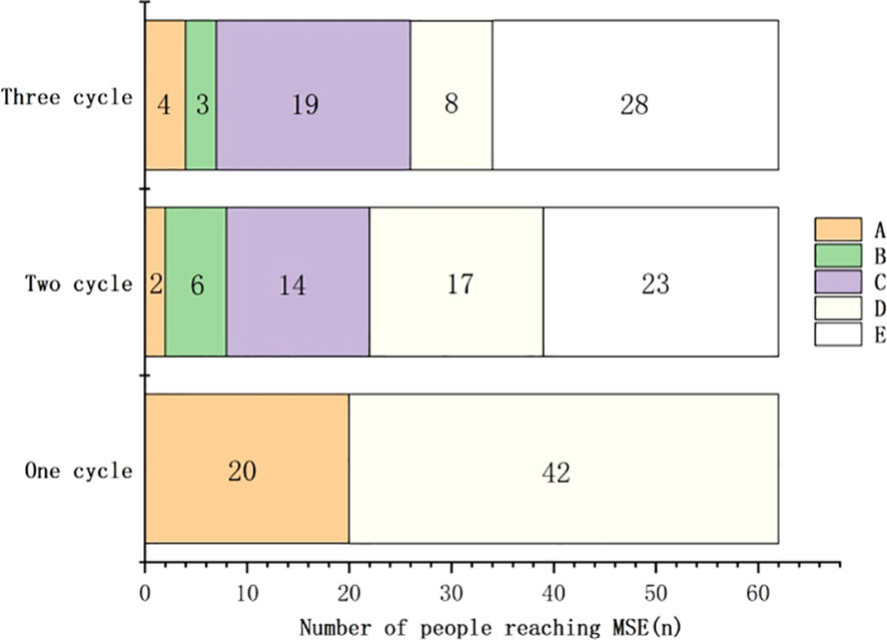
Changes in the number of patients achieving MSE, minimum symptom expression among 62 patients with VLOMG, very late-onset myasthenia gravis. The treatment with Efgartigimod resulted in a gradual increase in the number of MG patients achieving MSE. (A) Number of patients who newly achieved MSE; (B) Number of patients continuing treatment after achieving MSE; (C) Number of patients discontinuing treatment after achieving MSE; (D) Number of patients continuing treatment without achieving MSE; (E) Number of patients discontinuing treatment for other reasons without achieving MSE.

The median follow-up duration for 62 patients with VLOMG was 14.0 (7.0–16.0) months, while the median time to achieve MSE was 1.0 (1.0–3.0) months. MSE was achieved by 20 (32.3%), 22 (35.5%), and 25 (41.9%) patients after 1, 2, and ≥ 3 treatment cycles, respectively (**Figure 1**).

### Changes in MG-ADL scores

At 1 week after efgartigimod treatment initiation, 29.0% (18/62) of patients with VLOMG achieved CMI in MG-ADL scores.

**Figure 2:**
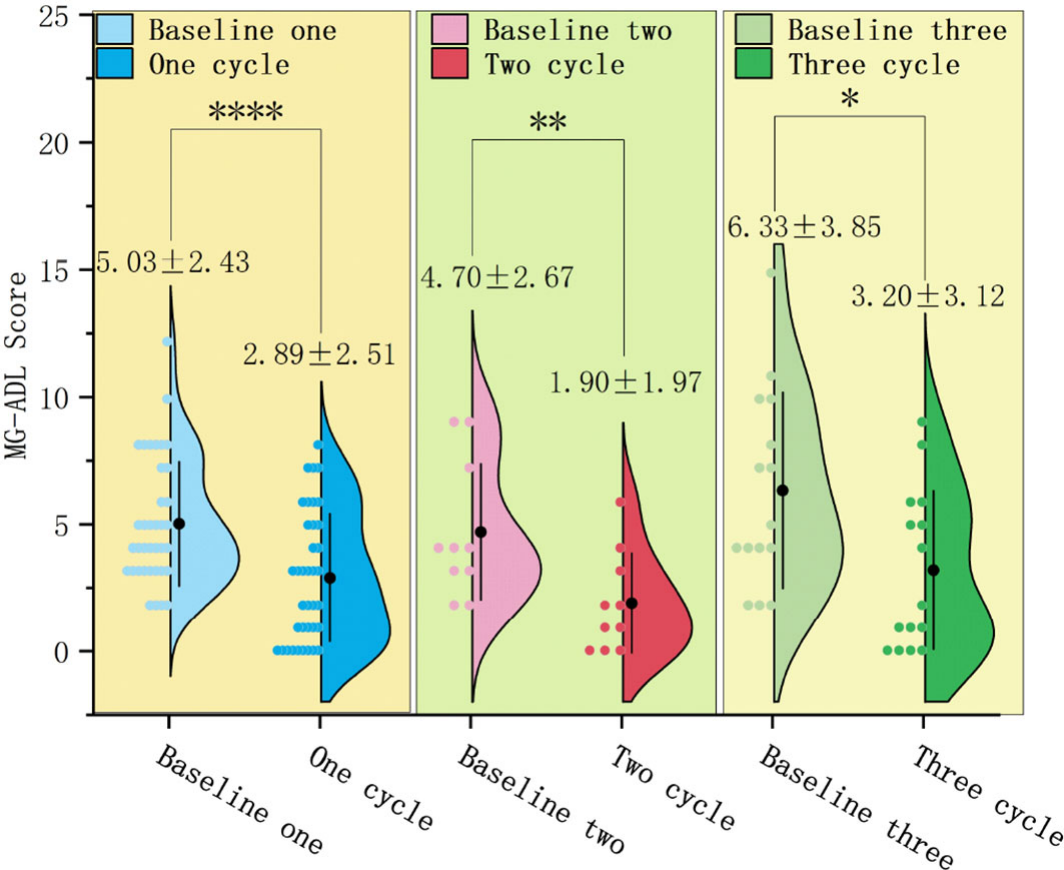
Changes in MG-ADL, Myasthenia Gravis-Activities of Daily Living scores in patients who underwent 1, 2, and ≥ 3 efgartigimod treatment cycles. The MG-ADL scores significantly reduced in patients undergoing 1 (^****^P < 0.0001), 2 (^**^P < 0.01), and ≥ 3 (P < 0.05) treatment cycles. Efgartigimod improves MG-ADL scores in patients during different treatment cycles.

**Figure 3:**
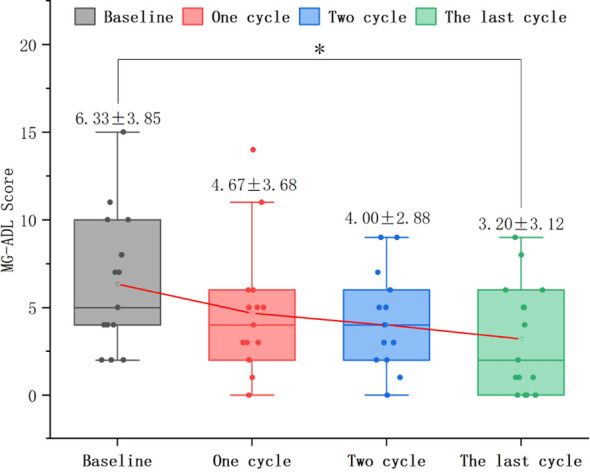
Dynamic changes in MG-ADL, Myasthenia Gravis-Activities of Daily Living scores in patients undergoing ≥ 3 efgartigimod treatment cycles. The MG-ADL scores after 1 and 2 treatment cycles were not significantly different from those at baseline (P = 0.458; P = 0.154). However, the MG-ADL score significantly decreased after the last cycle (P < 0.05). Efgartigimod reduces MG-ADL scores in the final cycle for patients with ≥3 treatment cycles.

- In 37 patients who underwent only 1 treatment cycle, the MG-ADL score after treatment (2.89 ± 2.51) significantly decreased relative to baseline (5.03 ± 2.43) (mean difference: 2.14 ± 2.80, T = 4.638, P < 0.0001). CMI was achieved by 23 patients (62.2%) (**Figure 2**).
- In 10 patients who underwent only 2 treatment cycles, the MG-ADL score after treatment (1.90 ± 1.97) significantly decreased relative to baseline (4.70 ± 2.67) (P < 0.01). CMI in MG-ADL scores was achieved by 8 patients (80.0%). Repeated measures analysis revealed a significant time effect with a gradual decrease in MG-ADL scores over time (F = 4.634, P < 0.05) (**Figure 2**).
- In 15 patients who underwent ≥ 3 treatment cycles, the MG-ADL score after treatment (3.20 ± 3.12) significantly decreased relative to baseline (6.33 ± 3.85) (P < 0.05). CMI in MG-ADL scores was achieved in 6 (40.0%), 8 (53.3%), and 11 (73.3%) patients after 1, 2, and final treatment cycles, respectively. Repeated measures analysis revealed a significant time effect with a gradual decrease in scores over time (F = 4.795, P < 0.01) (**Figures 2**, **3**).

**Figure 4:**
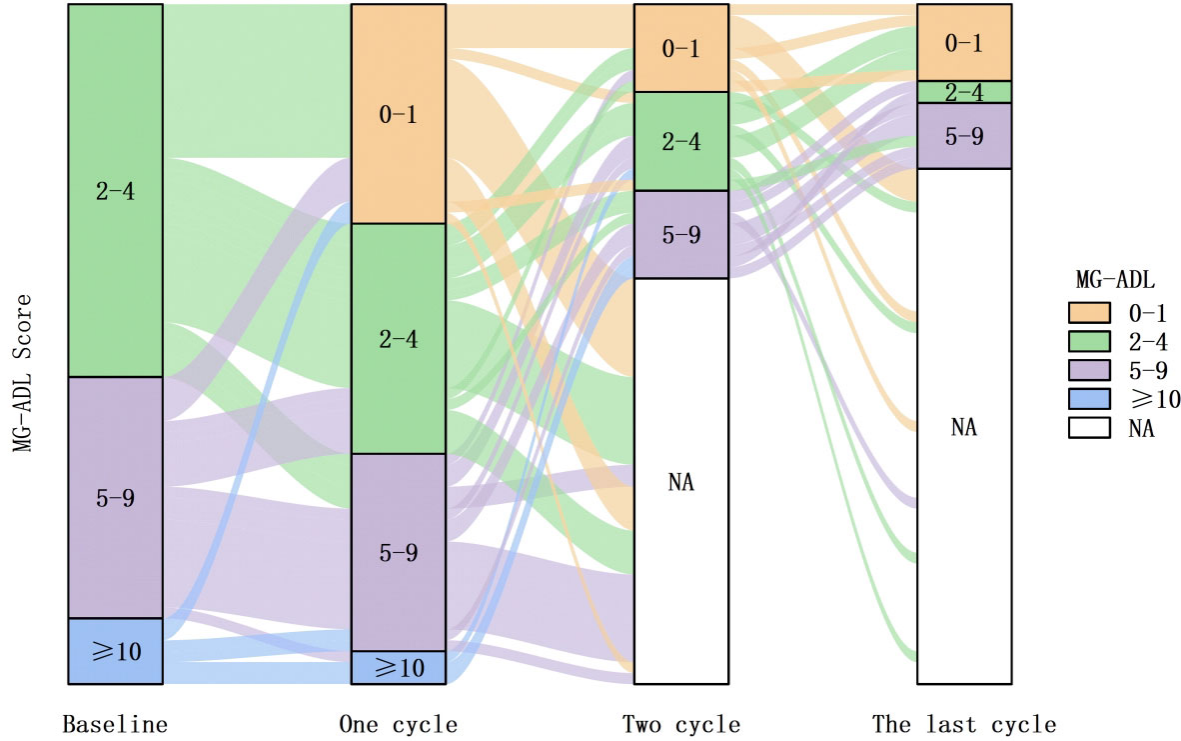
Distribution of MG-ADL, Myasthenia Gravis-Activities of Daily Living scores strata among 62 patients with VLOMG, very late-onset myasthenia gravis at baseline and after 1, 2, and ≥ 3 efgartigimod treatment cycles(NA indicates patients who did not continue treatment). Efgartigimod led to a shift in patients’ overall MG-ADL scores towards lower levels.

Stratification according to baseline MG-ADL scores demonstrated that all 62 patients shifted to a lower severity strata after efgartigimod treatment. The proportion of patients with severe MG (MG-ADL ≥ 10) decreased from 9.7% at baseline to 4.8%, 0.0%, and 0.0% after 1, 2, and ≥ 3 treatment cycles, respectively. Meanwhile, the proportion of patients with MG-ADL scores of 5–9 decreased from 35.5% at baseline to 9.7% after ≥ 3 treatment cycles (**Figure 4**).

### Changes in QMG scores

At week 1 post-efgartigimod treatment initiation, 31 of the 62 patients with VLOMG (50.0%) achieved CMI in QMG scores.

**Figure 5:**
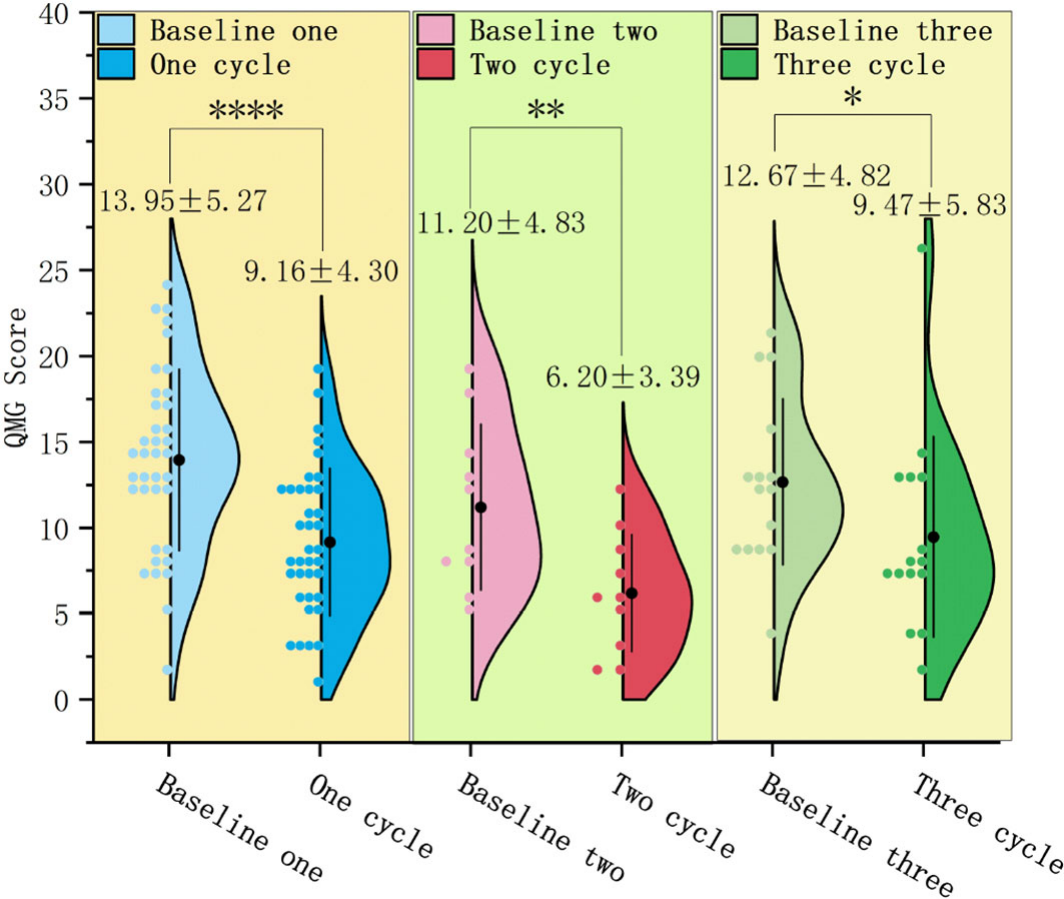
Changes in QMG, quantitative myasthenia gravis scores in patients who underwent 1, 2, and ≥ 3 efgartigimod treatment cycles. The QMG scores significantly decreased in patients who underwent 1 (^****^P < 0.0001), 2 (^**^P < 0.01), and ≥ 3 (P < 0.05) treatment cycles.Efgartigimod improves QMG scores in patients during different treatment cycles.

**Figure 6:**
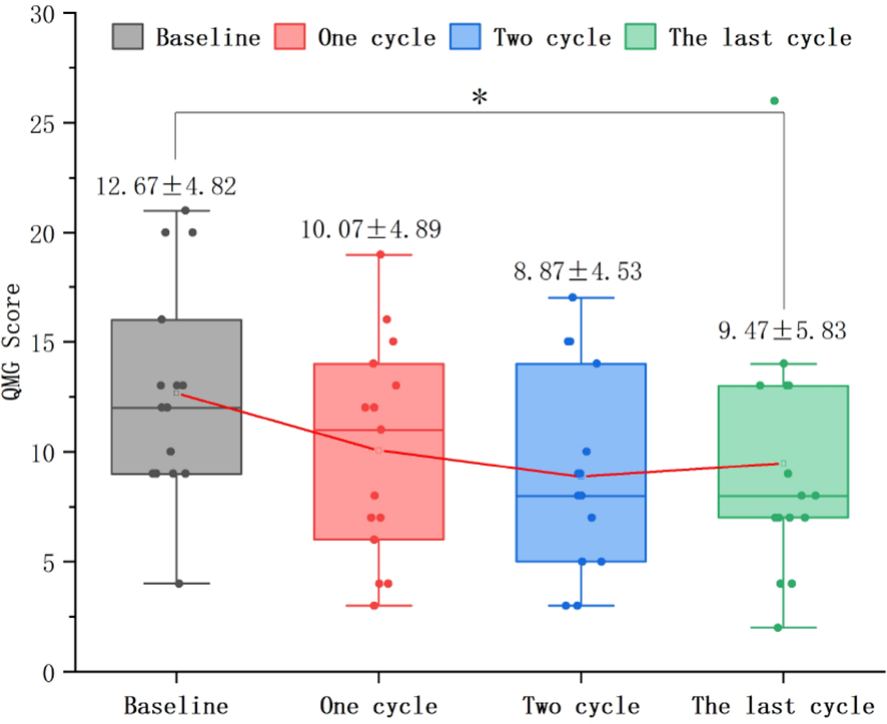
Dynamic changes in QMG, quantitative myasthenia gravis scores in patients who underwent ≥ 3 efgartigimod treatment cycles. The QMG scores after 1 and 2 treatment cycles were not significantly different from those at baseline (P = 0.264; P = 0.109). However, the QMG score significantly reduced after the last cycle (P < 0.05).Efgartigimod reduces QMG scores in the final cycle for patients with ≥3 treatment cycles.

- In 37 patients who underwent only 1 treatment cycle, the QMG score after treatment (9.16 ± 4.30) significantly reduced relative to baseline (13.95 ± 5.27) (mean difference: 4.78 ± 4.49, T = 6.479, P < 0.0001). CMI in QMG scores was achieved by 28 patients (75.7%) (**Figure 5**).
- In 10 patients who underwent only 2 treatment cycles, the QMG score after treatment (6.20 ± 3.39) significantly decreased relative to baseline (11.20 ± 4.83) (P < 0.01). CMI in QMG scores was achieved by 8 patients (80.0%). Repeated measures analysis revealed a significant time effect with a gradual decrease in QMG scores over time (F = 5.143, P < 0.05) (**Figure 5**).
- In 15 patients who underwent ≥ 3 treatment cycles, the QMG score after treatment (9.47 ± 5.83) significantly decreased relative to baseline (12.67 ± 4.82) (P < 0.05). CMI in QMG scores was achieved by 6 (40.0%), 8 (53.3%), and 8 (53.3%) patients after 1, 2, and ≥ 3 treatment cycles, respectively. Repeated measures analysis revealed a significant time effect with a gradual decrease in scores over time (F = 3.490, P < 0.05) (**Figures 5**, **6**).

**Figure 7:**
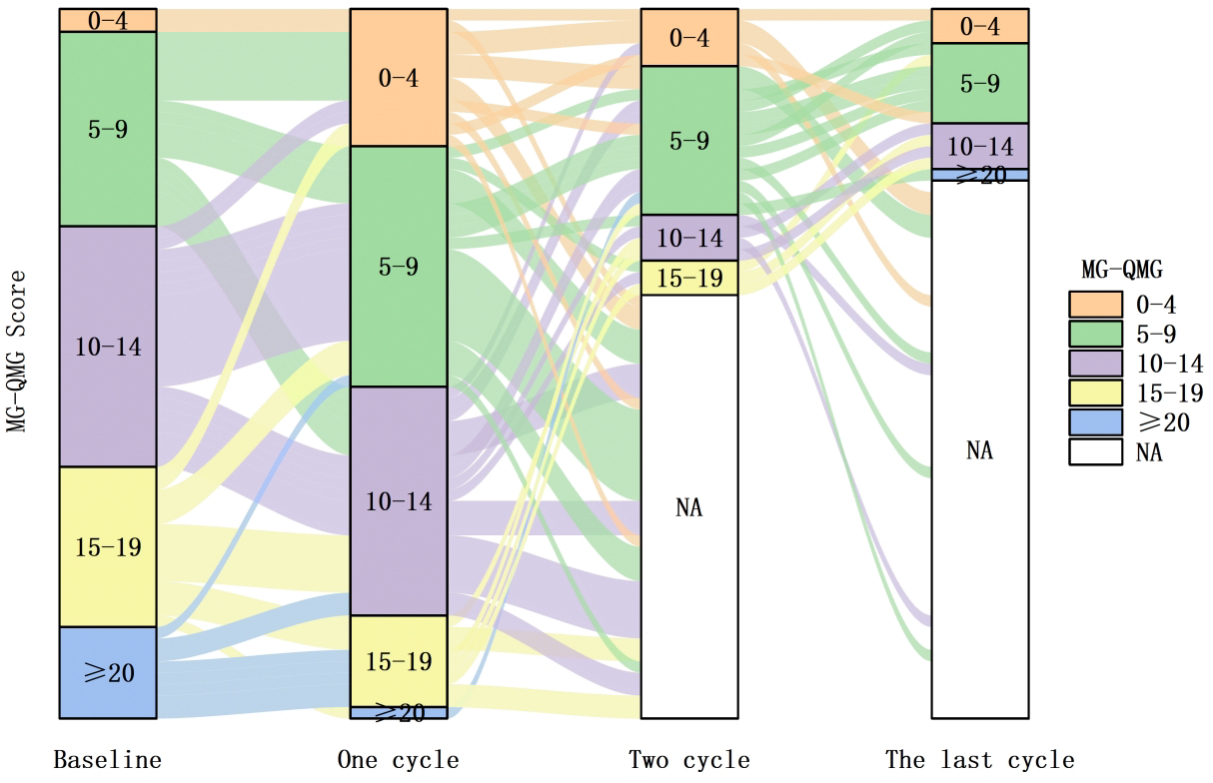
Distribution of QMG, quantitative myasthenia gravis scores strata among 62 VLOMG patients at baseline and after 1, 2, and ≥ 3 efgartigimod treatment cycles(NA indicates patients who did not continue treatment). Efgartigimod led to a shift in patients’ overall QMG scores towards lower levels.

Stratification by baseline QMG scores revealed a shift to a lower severity strata with a gradual time-dependent decrease in all 62 patients. The proportion of patients with severe MG (QMGs ≥ 20) decreased from 12.9% at baseline to 1.6%, 0.0%, and 1.6% after 1, 2, and ≥ 3 treatment cycles, respectively. Meanwhile, the proportion of patients with QMG scores of 15–19 decreased from 22.6% at baseline to 0.0% after ≥ 3 treatment cycles (**Figure 7**).

### Changes in MG-QoL15r scores

In 62 patients with VLOMG, the MG-QoL15r score after treatment (11.79 ± 6.99) significantly decreased relative to baseline (19.71 ± 10.83) (mean difference: 7.92 ± 8.61, T = 7.240, P < 0.0001).

**Figure 8:**
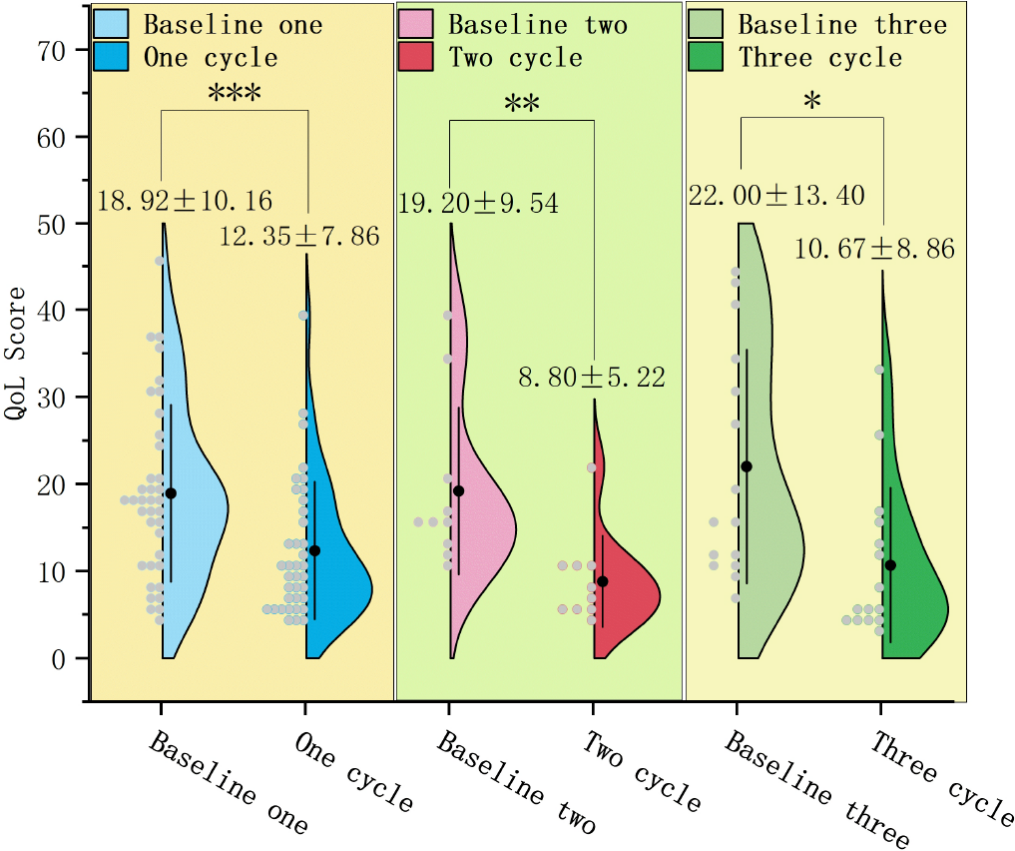
Changes in MG-QoL15r, myasthenia gravis-quality of life 15-item revised scores in patients who underwent 1, 2, and ≥ 3 efgartigimod treatment cycles. The MG-QoL15r scores significantly reduced in patients who underwent 1 (P < 0.001), 2 (P < 0.01), and ≥ 3 (P < 0.05) treatment cycles.Efgartigimod improves MG-QoL15r scores in patients during different treatment cycles.

**Figure 9:**
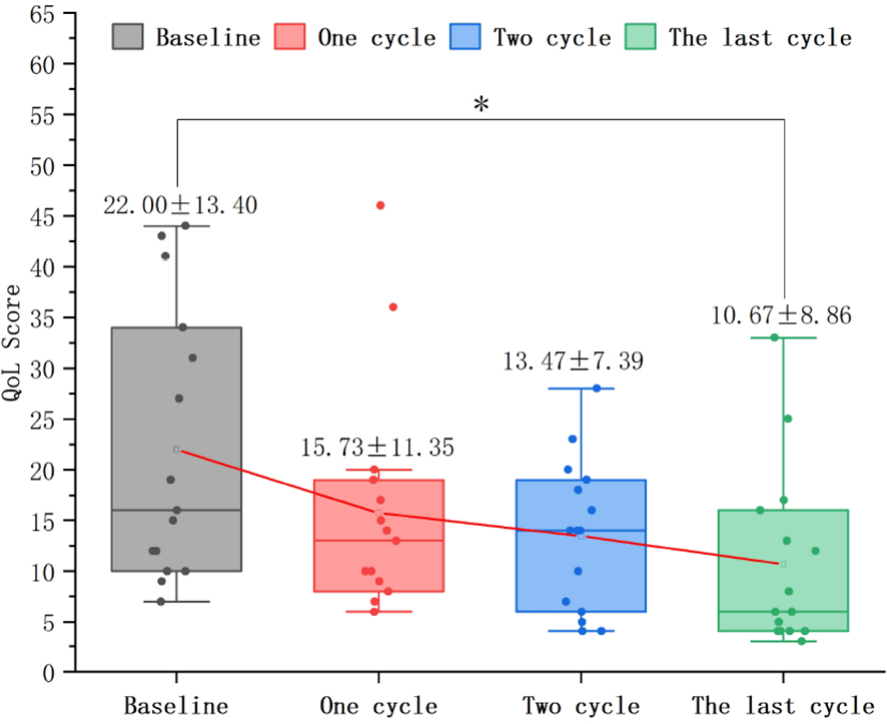
Dynamic changes in MG-QoL15r, myasthenia gravis-quality of life 15-item revised scores in patients who underwent ≥ 3 efgartigimod treatment cycles. The MG-QoL15r scores after 1 and 2 treatment cycles were non-significantly different from those at baseline (P = 0.416; P = 0.170). A significant reduction in the MG-QoL15r score was noted after the last cycle (^*^P < 0.05).Efgartigimod reduces QMG scores in the final cycle for patients with ≥3 treatment cycles.

- In 37 patients who underwent only 1 treatment cycle, the MG-QoL15r score after treatment (12.35 ± 7.86) significantly reduced relative to baseline (18.92 ± 10.16) (mean difference: 6.57 ± 9.94, T = 4.020, P < 0.001) (**Figure 8**).
- In 10 patients who underwent only 2 treatment cycles, the MG-QoL15r score after treatment (8.80 ± 5.22) significantly reduced relative to baseline (19.20 ± 9.54) (P < 0.01). Repeated measures analysis revealed a significant time effect with a gradual decrease in scores over time (F = 8.053, P < 0.01) (**Figure 8**).
- In 15 patients who underwent ≥ 3 treatment cycles, the MG-QoL15r score after the last treatment cycle (10.67 ± 8.86) significantly reduced relative to baseline (22.00 ± 13.40) (P < 0.05). Repeated measures analysis revealed a significant time effect with a gradual decrease in scores over time (F = 5.637, P < 0.01) (**Figure 9**).

### Changes in key muscle group function

In 37 patients who underwent only 1 treatment cycle, the scores of bulbar, limb (including axial), and ocular muscles after treatment (0.00 (0.00–1.00), 6.32 ± 2.60, and 2.00 (0.00–3.00), respectively) significantly improved relative to baseline (1.00 (0.00–2.50), 8.89 ± 3.81, and 3.00 (3.00–4.50), respectively) (median difference for bulbar muscles: 0.00 (0.00–1.00), Z = −2.861, P < 0.01; mean difference for limb muscles: 2.57 ± 2.94, T = 5.313, P < 0.0001; median difference for ocular muscles: 2.00 (0.00–3.00), Z = −3.528, P < 0.001).

In 10 patients who underwent only 2 treatment cycles, the ocular muscle score after treatment (0.80 ± 0.42) significantly improved relative to baseline (3.60 ± 0.50) (P < 0.01). Repeated measures analysis revealed a significant time effect with gradual improvement in ocular muscle function over time (F = 11.862, P < 0.001).

**Figure 10:**
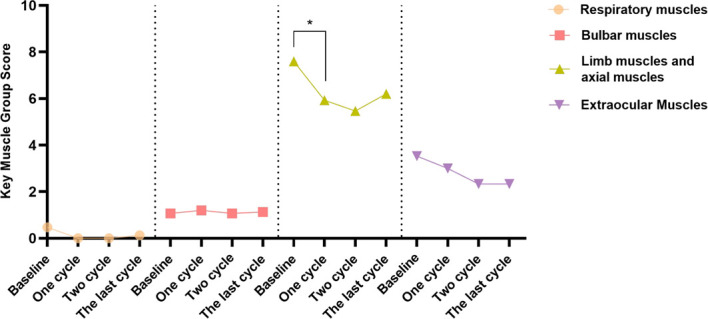
Changes in key muscle group function in patients who underwent ≥ 3 efgartigimod treatment cycles. A significant improvement in limb (including axial) muscle function was observed after 1 treatment cycle (^*^P < 0.05). Efgartigimod may improve limb muscle function in MG patients.

In 15 patients who underwent ≥ 3 treatment cycles, the limb (including axial) muscle score after 1 treatment cycle (6.20 ± 0.87) significantly improved relative to baseline (7.60 ± 0.85) (P < 0.05). Repeated measures analysis revealed a significant time effect with gradual improvement in limb muscle function over time (F = 4.280, P < 0.05) (**Figure 10**).

Efgartigimod treatment ameliorated symptoms of key muscle groups in patients with MG aged ≥ 65 years, especially limb (including axial) muscle symptoms.

### Dosage changes of conventional immunotherapies after efgartigimod treatment

In patients with VLOMG receiving glucocorticoid treatment, the median time to achieve a low dosage (≤ 5 mg) after efgartigimod treatment was 1.5 months (1.0–3.0 months) with an achievement rate of 68.8% (22/32).

**Figure 11:**
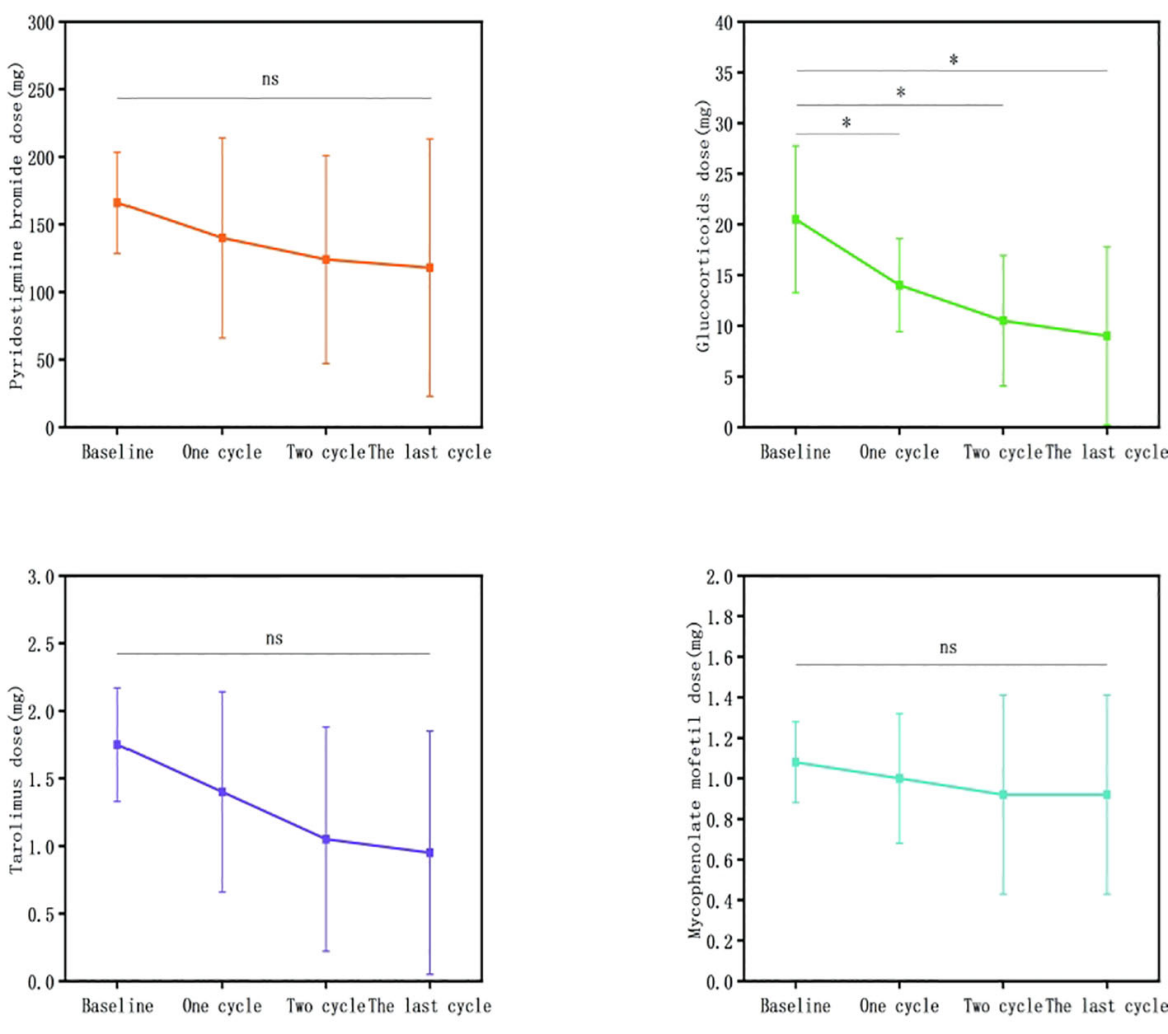
Changes in conventional immunotherapy dosages in patients who underwent ≥ 3 efgartigimod treatment cycles. Compared with those at baseline, glucocorticoid dosages decreased after 1, 2 and ≥ 3 treatment cycles. Pyridostigmine bromide and mycophenolate mofetil dosages were not significantly different before and after treatment (ns, ^*^P > 0.05).Efgartigimod may reduce the dosage of corticosteroids required for patients.

- In 37 patients who underwent only 1 treatment cycle, the dosages of glucocorticoids significantly decreased relative to baseline (mean difference: 13.82 ± 6.69, T = 9.007, P < 0.0001), pyridostigmine bromide (mean difference: 57.50 ± 91.66, T = 3.764, P < 0.001), tacrolimus (mean difference: 0.83 ± 1.02, T = 4.369, P < 0.0001), and mycophenolate mofetil (mean difference: 0.46 ± 0.66, T = 2.521, P < 0.05).
- In 10 patients who underwent only 2 treatment cycles, the baseline glucocorticoid dosage after treatment (0.00 ± 0.00 mg) significantly decreased relative to baseline (16.67 ± 2.89 mg) (P < 0.05). Meanwhile, the mycophenolate mofetil dosage after treatment (0.31 ± 0.59 g) significantly decreased relative to baseline (1.13 ± 0.23 g) (P < 0.05). The dosages of pyridostigmine bromide (P = 0.585) and tacrolimus (P = 0.136) did not significantly vary before and after treatment. Repeated measures analysis revealed significant time effects with gradual reductions in glucocorticoid, tacrolimus, and mycophenolate mofetil dosages over time (F = 76.000, P < 0.05; F = 5.813, P < 0.05; F = 8.795, P < 0.01).
- In 15 patients who underwent ≥ 3 treatment cycles, the glucocorticoid dosage after treatment (9.00 ± 8.76 mg) significantly decreased relative to baseline (20.50 ± 7.25 mg) (P < 0.05). The dosages of pyridostigmine bromide (P = 0.505), tacrolimus (P = 0.080), and mycophenolate mofetil (P = 1.000) were not significantly different before and after treatment. Repeated measures analysis revealed significant time effects with gradual reductions in glucocorticoid and tacrolimus dosages over time (F = 12.787, P < 0.01; F = 6.531, P < 0.05) (**Figure 11**).

### Safety of efgartigimod

Serious adverse events, deaths, or severe infections were not recorded during the follow-up period. Only one patient with VLOMG discontinued efgartigimod due to an allergic rash, and three patients experienced mild adverse reactions associated with efgartigimod administration continued therapy, including one case of gastric discomfort and two cases of headache.

## Discussion

In the last 120 years, the mortality rate of MG has decreased from nearly 100% to ≤ 5%. The prevalence of MG has increased significantly from 1 case per 200,000 people to 1 case per 1,800 people during the same period (10). In particular, the prevalence of MG has increased in elderly patients aged ≥ 50 years, which may be attributed to improved epidemiological methods, advances in diagnosis, global population aging, and immunosenescence (7, 21). In this study, 69.0% (746/1108) and 32.2% (327/1108) of patients presented with LOMG (≥ 50 years) and VLOMG (≥ 65 years), respectively. Conventional non-specific immunosuppressive therapies for LOMG are associated with several limitations, including slow onset of action, extensive side effects, and increased risks of infection, hypertension, and diabetes mellitus with long-term use, impairing physiological immune function. These effects are severe in patients with LOMG (22) and impose strict requirements for medication selection in patients with VLOMG aged ≥ 65 years with a frail baseline status. Thus, there is an urgent need to identify therapeutics with a favorable risk-benefit ratio.

Recently, the emergence of biological targeted agents has addressed these unmet needs. Efgartigimod, an FcRN-binding biological targeted inhibitor, rapidly downregulates pathogenic IgG antibody levels through unique mechanisms of action, rapidly alleviates severe symptoms, and consequently improves the quality of life and decreases the need for intensive care. However, evidence for the efficacy of efgartigimod in patients aged ≥ 65 years is limited in the phase III ADAPT trial (which evaluated the efficacy and safety of efgartigimod in generalized MG patients aged ≥ 18 years) and some real-world studies (16, 23, 24), leading to clinical uncertainties regarding its use in this age group.

In this study, most patients achieved CMI in MG-ADL (54.8%) and QMG (62.9%) scores after one efgartigimod treatment cycle. In the ADAPT trial, CMI achievement rates in MG-ADL and QMG scores were 68% and 63%, respectively. The lower CMI rate in MG-ADL scores in this study can be attributed to the poor baseline clinical status (MGFA Grade IV: 21.0% vs. 4% in ADAPT) and the study population characteristics. Additionally, MG-ADL items, such as rising from a chair and shortness of breath after activity, may be affected by other comorbidities (including cerebrocardiovascular disease). This study demonstrated that efgartigimod rapidly alleviates symptoms in patients with VLOMG. Approximately one-third of patients with VLOMG exhibited symptom improvement at week 1 post-first administration (CMI rates in MG-ADL and QMG scores were 29.0% and 50%, respectively). Although the time window to achieve the optimal efficacy varied among individuals, this process was similar to that in younger populations in other studies, with no significant delay (18). This rapid onset of action of efgartigimod provides a therapeutic advantage for elderly patients in urgent need of severe symptom control.

The cumulative MSE achievement rate in 62 patients with VLOMG increased gradually from 32.3% (20/62) after 1 treatment cycle to 41.9% (26/62) at the last follow-up, which is comparable to the 40% MSE achievement rate in the ADAPT trial. Furthermore, the number of patients with VLOMG achieving MSE increased with prolonged efgartigimod treatment. After ≥ 3 treatment cycles, 4 additional patients achieved MSE. The MG-ADL, QMG, and MG-QoL15r scores significantly decreased after the last treatment cycle in 15 patients who underwent ≥ 3 treatment cycles. The same indicators significantly improved in 10 patients who underwent only 2 treatment cycles with significant time effects, indicating gradual improvement with prolonged treatment. In the ADAPT trial, the CMI rate in MG-ADL scores increased from 68% after the first treatment cycle to 78% after subsequent treatment cycles. The mean improvement in MG-ADL and QMG scores after the second treatment cycle in the efgartigimod group was higher than that in the placebo group.

The MG-QoL15r scores after treatment significantly decreased relative to baseline at the end of follow-up in both patients who discontinued treatment and those who continued treatment. Efgartigimod maintains the quality of life of patients with VLOMG. Although MG-QoL15r scores gradually decreased over time in 15 patients who underwent ≥ 3 treatment cycles, the difference was significant after the last cycle. In the ADAPT trial, the difference was significant at week 1 post-treatment initiation, with the maximum improvement at week 5. This discrepancy may be due to the high burden of comorbidities in the study cohort (all aged ≥ 65 years), which may lead to an inaccurate subjective perception of MG symptom improvement and consequently affect MG-QoL15r scores. Thus, efgartigimod exhibits stable efficacy in patients with VLOMG with complex clinical conditions and can continuously improve their quality of life with multiple treatment cycles.

Quality of life improvement is a key therapeutic goal for elderly patients, encompassing multiple dimensions, including symptom relief, treatment burden reduction, and minimization of side effects.

Improving the muscle group function enhances the quality of life. The hospitalization and mortality rates are high among patients with VLOMG due to the risks of dysphagia, dysarthria, and respiratory failure (25). Efgartigimod alleviated bulbar and respiratory muscle weakness, a common life-threatening symptom in patients aged ≥ 65 years. This study confirmed that efgartigimod effectively and significantly improved limb (including axial) and ocular muscle function but not respiratory muscle function. The respiratory muscle scores after treatment non-significantly decreased relative to baseline in all treatment cycle groups. This may be attributed to the relatively good baseline status of respiratory muscles with limited room for improvement. Limb (including axial) muscle function in 15 patients who underwent ≥ 3 treatment cycles slightly worsened after the second and last cycles, which may be due to the inherent symptom fluctuation of MG and the superimposed effects of other age-related comorbidities.

Decreased treatment burden enhances the quality of life. Glucocorticoids are the first-line immunosuppressive therapy for all MG subgroups (26). However, glucocorticoids are associated with frequent and extensive side effects, such as osteoporosis, weight gain, hypertension, and glucose intolerance. Some of these side effects further exacerbate common comorbidities in patients with VLOMG. Zhang Z reported a significant reduction in the usage of conventional immunosuppressants, including glucocorticoids (from 46.7% to 13.3%), after 5 weeks of treatment (19). In our study, the dosages of glucocorticoids, pyridostigmine bromide, tacrolimus, and mycophenolate mofetil were reduced in patients who underwent only 1 efgartigimod treatment cycle. However, the dosage reduction was not significant for some drugs in patients who underwent 2 and ≥ 3 treatment cycles. The dosages of glucocorticoids were significantly reduced in all our groups. These findings indicate that efgartigimod can mitigate the adverse effects of conventional immunosuppressants, especially glucocorticoids, which exacerbate or induce comorbidities.

Favorable safety profile safeguards quality of life. Only 5% of patients experienced serious adverse events in the ADAPT trial. And in our study, no serious adverse events, deaths, or MG exacerbation/infection-related crises were noted during efgartigimod treatment. Only one patient discontinued treatment due to a rash, while three patients with mild adverse reactions associated with efgartigimod administration continued therapy. These findings indicate that efgartigimod maintains a favorable safety and efficacy profile in patients with VLOMG (11). This study further verified that efgartigimod improved the quality of life of elderly patients, especially parameters related to muscle function, medication optimization, and safety.

Two special cases of super-elderly patients (> 85 years) exhibited long-term stable efficacy or favorable safety with efgartigimod treatment. The first patient was an 89-year-old male with a 4-year history of MG complicated with hypertension, ischemic cerebrovascular disease, and sleep disorders. This patient exhibited an inadequate response to long-term treatment with pyridostigmine bromide and tacrolimus. Efgartigimod treatment was initiated in April 2024. The patient achieved CMI in MG-ADL and QMG scores after 4 weeks, as well as in MSE after 6 months. Maintenance treatment was continued for 18 months with improved quality of life and tacrolimus discontinuation. The second patient was an 87-year-old male with a 1-year history of MG complicated with hypertension, coronary atherosclerotic heart disease, and emphysema. Efgartigimod treatment was initiated in November 2024 and discontinued after 4 months. No significant symptom improvement was observed during treatment. However, serious adverse events did not occur, confirming a favorable safety profile.

At the study center, 5 and 7 patients discontinued multiple efgartigimod treatment cycles due to symptom exacerbation and no significant improvement, respectively. This is a multifactorial outcome influenced by efficacy, safety, economic burden, and treatment convenience. Economic burden is the main influencing factor. Efgartigimod is a novel targeted biological agent for MG and is expensive. Even with medical insurance coverage, low reimbursement rates, restrictions on off-site medical treatment, and strict renewal criteria prevent many patients who achieved MSE (and desired maintenance or further improvement) from continuing treatment due to unaffordable costs. Efgartigimod therapy involves regular intravenous infusions at designated medical centers, imposing a burden on patients living in remote areas or those with limited mobility (especially the elderly). Additionally, patients with VLOMG often have multiple chronic diseases (such as tumors, chronic infections, and cerebrocardiovascular diseases). The complex systemic status of these patients may affect the response to immunomodulatory therapy (27–29). Long-term use of prior potent immunosuppressants may alter the immune microenvironment. Furthermore, frequent comorbidities may also impact the efficacy of efgartigimod (30, 31).

This study has several limitations. In this study, more than half of patients with VLOMG underwent only 1 treatment cycle, while a small number of patients underwent 2 or ≥ 3 treatment cycles. The number of patients receiving multiple cycles will be expanded in the future to accurately and rigorously evaluate the efficacy and safety of multi-cycle efgartigimod regimens. The placebo effect or the long-term effects of prior treatment regimens cannot be completely excluded. Multiple comorbidities and polypharmacy prescriptions are common in patients with VLOMG. Thus, drug-drug interactions may affect the efficacy of efgartigimod. Long-term studies and monitoring are required with a focus on cerebrocardiovascular disease and long-term infection risks.

## Conclusions

This study demonstrated the therapeutic efficacy of efgartigimod in VLOMG. Efgartigimod effectively ameliorates symptoms, improves quality of life, and enables short-term glucocorticoid dosage reduction in patients with VLOMG. Thus, efgartigimod seems to be a safe and effective long-term therapeutic agent for VLOMG.

## Data Availability

The original contributions presented in the study are included in the article/supplementary material. Further inquiries can be directed to the corresponding author.
